# The effect of sub-clinical infection with *Mycobacterium avium* subsp*. paratuberculosis* on milk production in a New Zealand dairy herd

**DOI:** 10.1186/s12917-018-1421-4

**Published:** 2018-03-14

**Authors:** Andrew Bates, Rory O’Brien, Simon Liggett, Frank Griffin

**Affiliations:** 1 Vetlife, Centre for Dairy Excellence, Wilson Street, Geraldine, New Zealand; 2 Disease Research Limited, Invermay Agricultural Centre, Mosgiel, New Zealand; 3 Department of Microbiology & Immunology, University of Otago, Dunedin, New Zealand

**Keywords:** Johne’s disease, Milk production, ELISA, Quantitative PCR

## Abstract

**Background:**

Johne’s disease is a major production limiting disease of dairy cows. The disease is chronic, progressive, contagious and widespread; there is no treatment and there is no cure. Economic losses arise from decreased productivity through reduced growth, milk yield and fertility and capital losses due to premature culling or death. This study attempts to address the effect of subclinical JD on milk production under New Zealand pastoral dairy farming conditions using a new testing approach. Blood samples were taken from all lactating animals from a single seasonally calving New Zealand dairy herd in the autumn of 2013 and 2014. Samples were subject to serological assay for antibodies to *Mycobacterium avium* subsp. *paratuberculosis* using a combination of four ELISA tests in parallel followed by selective quantitative fecal PCR to confirm the fecal shedding characteristics of ELISA positive cows. ELISA status was classified as Not-Detected, Low, Moderate or High and fecal PCR status as Not-Detected, Moderate or High.

**Results:**

A mixed generalized regression model indicated that, compared to cows where MAP was not detected, daily milk solids production was 4% less for high ELISA positive cows (*p* = 0.004), 6% less for moderate fPCR cows (*p* = 0.036) and 12% less for high fPCR cows (*p* < 0.001).

**Conclusions:**

This study confirms that sub-clinical JD can have a significant impact on milk production and that the testing methodology used stratified the animals in this herd on their likely impact on production and disease spread. This allowed the farmer to prioritize removal of heavily shedding, less-productive animals and so reduce the risk of infection of young stock. This is the first longitudinal study based in New Zealand looking at the effect of Johne’s infection status on daily milk production allowing for intermediary and confounding factors.

## Background

Johne’s disease (JD) is a chronic disease of ruminants caused by intestinal infection with *Mycobacterium avium* subsp. *paratuberculosis* (MAP). Johne’s infection is predominantly subclinical in most dairy cows with farmers becoming aware of the disease when the clinical signs of infection such as diarrhoea and wasting become apparent [1]. Whitlock and Buergelt [2] suggested a bovine JD “iceberg effect” whereby, for every clinically affected animal born on the farm, a minimum of 25 other animals are likely to be infected.

Many studies have found MAP infection to be associated with a statistically significant reduction in milk production [3–6], although this depends on the cow’s age [3], farm system [7], the genetics of the cow [8] and strain of MAP [9]. However, few studies have examined the effect of MAP infection on milk production under seasonal, pastoral systems in NZ. Comparing differences in milk production between MAP positive and negative cows is not straight forward because of differences in quantification of milk yield, trial design, diagnostic tests, farm system and the presence of confounding and intermediary factors [6, 10].

Farmer perception has a major impact on the uptake of disease control schemes and engagement at the individual farm level [11]. Many farmers without direct experience of clinical JD in their herds consider that JD is not a major problem for their farm. Norton et al. [12] found only 10% (22/225) of NZ farmers that had not observed clinical cases in their herd considered the disease a serious problem. Similar findings were reported by Sorge et al. [13] in a Canadian survey where most dairy farmers did not consider JD a serious problem for their operation. Without clear evidence that MAP infection is linked to decreased milk production, the low clinical incidence of JD in most herds allows farmers to relegate MAP infection to the realm of natural losses.

The specificity of ELISA tests may be compromised by common antigens shared between MAP, *Mycobacterium avium* and other saprophytic environmental mycobacteria. The sensitivity of ELISA tests, particularly for sub-clinically infected animals in the early stages of JD, is also influenced by the dynamics of antibody production [14]. While detection of the organism via fecal culture on Herrold’s egg yolk medium has been a definitive test for MAP infection this requires prolonged incubation periods of up to 16 weeks and may be compromised by overgrowth by contaminating gut organisms [15–17]. Internationally, the rapid, direct and quantitative measurement of MAP shedding in feces of infected and affected animals by quantitative PCR is rapidly becoming a standard and widely used method for JD diagnostic testing [18–20].

As no single diagnostic test satisfies all criteria in terms of sensitivity, specificity, speed of turnaround, cost and convenience, combinations of tests are used to achieve optimal diagnosis [21, 22]. In NZ, a herd testing protocol based on an initial herd screening with a serological ELISA for multiple MAP antigens (Paralisa™) [23] coupled with a quantitative fecal PCR (fPCR) test to confirm the status of ELISA positive animals [24] has been developed. This allows farmers and their advisers to stratify shedders according to disease status and environmental risk. However, the impact of subclinical infection, as defined by this test, has not yet been characterized for milk solids production under NZ pastoral systems.

The present longitudinal study attempted to quantify the effect of MAP infection on individual lactation test day milk production on a single, seasonally calving, NZ pastoral dairy herd allowing for potential confounders.

## Methods

### Study animals

A spring calving, pasture based, Friesian dairy herd (1250 cows at peak milk) in the South Canterbury region of NZ was selected for the study. In the 5 years preceding this study, the herd had culled 3–5% of the milking herd annually from suspected clinical JD based on clinical signs observed by the owner. In 2009, MAP had been isolated, and JD confirmed histopathologically, from gut and mesenteric lymph node samples from each of 4 cull animals suspected of clinical JD. One year later, all milking cows over 2 years old were subject to a single serologically based ELISA (Paralisa™) with fPCR performed on a small subset of the ELISA positive animals. At this test, 97/1086 (8.93%) were ELISA positive and approximately 20% of these ELISA positive cows were shedding high levels (exceeding ≥1 × 10^4^ genomes/mL) of MAP as determined by fPCR. Considering the high prevalence of ELISA-positive animals the farmer was reluctant to cull all seropositives, most of which appeared healthy and productive. Persistent losses (> 3% pa) of clinically affected animals continued from 2010 to 2013. In 2014 a decision was made to rescreen the herd using serial ELISA and fPCR testing to identify animals which were shedding high levels of MAP, for future culling.

### Sample acquisition and treatment

In the autumn of 2014 and again in the autumn of 2015, a coccygeal tail vein blood sample was collected into a plain blood tube from all milking cows in the enrolled herd. Samples were transported to DRL (Disease Research Ltd, Mosgiel, NZ) and assayed for circulating antibody to MAP by serum ELISA using a combination of two ELISA tests, Paralisa™ (DRL, Mosgiel, NZ) and IDEXX Paratuberculosis Screening Ab Test (IDEXX Laboratories, Inc., Westbrook, ME, USA). The Paralisa™ methodology was based on previously published procedures for ELISA immunoassays used to diagnose immune reactions to MAP infection in farmed red deer [23, 25]. In addition to the IgG_1_ antibody responses to a denatured antigen in the form of Purified Protein Derivative J (Johnin) and a native protein in the form of Protoplasmic Antigen (PPA), in this study an additional MAP-specific recombinant protein antigen, Ag_1_Del_1_, was incorporated into the Paralisa™ test protocol. Final test results were arrived at by considering the antibody level to the IDEXX test and the three Paralisa™ test antigens in parallel. IDEXX ELISA assays were performed and interpreted according to the instructions supplied by the kit manufacturer. Results were classified as follows; for the Paralisa™ serological assays, a classification of Not Detected was returned for results of < 50 ELISA Units (EU) for Johnin, PPA, and Ag_1_Del_1_ antigens, readings of 50–100 EU in any one test were classified as Low, readings of 101–150 EU as Moderate, and readings of > 150 EU as High. For the IDEXX tests, results were classified as Not Detected (S/*P* < 55), Low (S/*P* ≥ 55), Moderate (S/*P* ≥ 100) or High (S/*P* ≥ 150) based on the response relative to the kit positive control.

Seven days later, a single fecal sample (10 g approx.) was collected from each cow testing Low, Moderate or High to any of the ELISA tests and forwarded to DRL for quantitation of MAP shedding in feces by fPCR [24, 26]. Quantitation of MAP DNA titer in fecal samples was accomplished using a standard curve comprising DNA dilution standards spanning 7 serial log dilutions of MAP genomic DNA prepared from MAP laboratory strain 316f and results extrapolated and reported as ‘MAP genome copies/mL’ equivalents. DNA standards ranged from 16.5 μg/mL to 1.65×10^− 5^ μg/mL; 3uL of DNA standard was utilized in each (20uL) PCR reaction such that, given a MAP genome size of 4.8Mbp [27], these values equated to a topmost standard of 1 × 10^7^ genomes/20uL (5 × 10^8^ genomes/mL) down to a lowermost standard of 10 genomes/20uL reaction (or 500 genomes/mL).

Fecal sample data were stratified into shedding categories with MAP shedding scores of ≥1 × 10^3^ to < 1 × 10^4^ genomes/mL classified as Moderate and counts exceeding ≥1 × 10^4^ genomes/mL as High. In this study, fecal samples which returned shedding scores of < 1 × 10^3^ genomes/mL feces were conservatively classified as Not Detected. Classification of MAP status by ELISA and fPCR results is summarized in Table 1.

**Table 1:** Classification scheme of MAP status from ELISA and fPCR results in a study looking at the effect of MAP infection on milk production in a South Canterbury dairy herd

Test				MAP status
ELISA				
Paralisa™			IDEXX^a^	
Johnin^a^	PPA^a^	Ag_1_Del_1_^a^		
< 50 EU	< 50 EU	< 50 EU	Not Detected	Not Detected
50–100 EU in any test			Low	Low
101–150 EU in any test			Moderate	Moderate
> 150 EU in any test			High	High
fPCR				
< 1×10^3^ genomes/mL				Not Detected
≥ 1×10^3^ - < 1 × 10^4^ genomes/mL				Moderate
≥ 1×10^4^ genomes/mL				High

^a^ELISA results were interpreted in parallel

^b^fPCR results were interpreted in series with ELISA results

### Milk production data

For each cow, milk production was measured at 4 herd test dates spread every 60–70 days throughout the lactation. At each herd test, individual cow production in kg milk solids (kgMS) and individual somatic cell count (ISCC) was recorded as cells/mL for the test day. Third-party electronic access to individual cow age, breed, calving date, milk quality and production data were granted.

### Statistical analysis

The outcome variable was level of kg milk solids, (continuous) recorded at each herd test day for the 2013–2014 season and for the 2014–2015 season. In the NZ seasonal dairying context, season refers to the period in milk from calving in the spring (July–August) to dry off in the autumn (April–May) and typically lasts 280 days. The predictor variables included how many days in milk from calving at test day (continuous), parity at sampling date, breed (categorized as > 75%, 50–75%, < 50% proportion of Friesian genetics), milking season (categorical; 2013–14 or 2014–15) and MAP infection status. Test day somatic cell linear score (LS, indicating the log_2_ of the SCC transformed as (log_2_(ISCC/100,000)) + 3 [28] was also included as a predictor variable. A separate time variable was created for milk test-day number indicating the number of milk test days for a given animal since the beginning of the lactation. Models were constructed with parity at sampling date treated as a continuous variable and also divided into primiparous and multiparous to account for differences in lactation shape [4, 5]. The model with the lowest value for Akaike Information Criteria (AIC) was selected.

ELISA test and fPCR fecal status as categorized above were used to describe MAP status modifying the methodology described by Smith et al. [4]. Five stages of MAP infection were identified: Undetected, Moderate Latent, High Latent, Moderate Shedding and High Shedding. The aim of the present study was to assess the relationship between test result and milk production in the current lactation. Given that cows were tested 60 days from the end of lactation and the greater sensitivity of the ELISA tests used, cows were assigned a MAP status based on the test results for the current lactation. Cows with a negative ELISA and fPCR result were classified as undetected. Cows with at least one positive test result were considered infected. Animals with a positive fPCR result were classified as shedding for all milk test days of the current lactation; moderate shedding if the fPCR ≥1 × 10^3^ to < 1 × 10^4^ genomes/mL and high shedding when fPCR ≥1 × 10^4^ genomes/mL. Cows with a positive ELISA result in the absence of a positive fPCR result were classified as latent infection. For all milk test days prior to a positive ELISA result cows were classified latent as it was assumed that cows were infected in calf-hood.

Database summaries and plots were used to explore the data. All variables were assessed for correlation using a correlation matrix and where a correlation > 0.2 was found, a variance inflation factor to assess collinearity was calculated using auxiliary regressions of one of the correlated variables on the remaining explanatory variables in the model. When the variance inflation factor was > 10, or if on rerunning the model without the variable the remaining coefficients changed in value by more than 20%, the collinear variables were assessed for biological plausibility. In this situation, the least useful variable was discarded from the final model using a Likelihood ratio test.

The effect of the input variables was investigated using pair-wise combinations in an ANOVA with a Bonferroni adjustment for multiple comparisons. Individual variables and their two-way interaction terms were carried forward to a multivariable linear regression model if ANOVA indicated the level of significance was < 0.1. Given that there were only a small number of variables a hand built model was constructed for the dependent variable (kg milk solids at each test day). Model structure was based on that of Smith et al. [4]. Age, MAP status, breed, days in milk and LS were all modelled as fixed effects. Given that there were only 2 seasons and the average number of lactations per cow was 1.51, milking season was modelled as a fixed effect with cow modelled as a random effect. To allow for correlation of test day results within individual cows, first order autoregression using test day number of a cow within lactation to identify the time lag between milk test day observations in individual cows was used. The model structure was:

$$ {\displaystyle \begin{array}{c}{\mathrm{kgMS}}_{\mathrm{i}\mathrm{lt}}={\upbeta}_0+{\upbeta}_{1,\mathrm{p}}\mathrm{parity}+{\upbeta}_2{\mathrm{DIM}}_{\mathrm{i}\mathrm{lt}}+{\upbeta}_3\exp \left[-0.1\kern0.5em \times \kern0.5em {\mathrm{DIM}}_{\mathrm{i}\mathrm{lt}}\right]+{\upbeta}_{4,\mathrm{b}}\mathrm{breed}\\ {}+{\updelta}_{1,\mathrm{p}}{\mathrm{DIM}}_{\mathrm{i}\mathrm{lt}\ \mathrm{x}}\mathrm{parity}+{\updelta}_{2,\mathrm{p}}\exp \left[-0.1\times {\mathrm{DIM}}_{\mathrm{i}\mathrm{lt}}\right]\times \mathrm{parity}\\ {}+{\upbeta}_{5,\mathrm{y}}\mathrm{season}+{\upbeta}_{6,\mathrm{n}}{\mathrm{MAP}}_{\mathrm{i}\mathrm{lt}}+\left({\uprho}_{\mathrm{i}}{\upvarepsilon}_{\mathrm{i},\mathrm{t}-1}+{\upmu}_{\mathrm{i}\mathrm{lt}\mathrm{pmn}}\right)\end{array}} $$

In this model, the outcome is daily milk production (in kgMS/day), *i* indicates cow, *l* indicates the present lactation, and *t* indicates milk test day; *β*_1,*p*_ is the fixed effect of the dichotomized *p*th parity (*p* = 1, > 1); *β*_2_ is the effect of Days in Milk (DIM); *β*_3_ is the effect of Wilmink’s correction; *β*_4,b_ is the effect of breed b = > 75%, 50–75%, < 50% Friesian genetics; *β*_5,*y*_ is the fixed effect of the *y*th season (*y* = 2013–14; 2014–15); and *β*_6,*n*_ is the fixed effect of the *n*th JD status (n = Undetected, Moderate-Latent, High-Latent, Low-Shedding, High-Shedding). The interaction coefficient *δ*_1,*p*_ is the effect of the interaction between DIM and the *p*th parity, and the interaction coefficient *δ*_2,*p*_ is the effect of the interaction between Wilmink’s correction and the *p*th parity [29]. The term *ρ*_*i*_*ε*_*i,t −* 1_ provides the first-order autoregression between milk test days in individual cows; and *u*_*iltpmn*_ is the error term for each test date [4].

A dependent variable such as milk production may be associated with many input variables which may in turn be associated with each other [30]. A causal web was used to understand the likely nature of the relationship between potential input variables and to allow for confounding and intermediary variables [6]. A confounding variable has to be associated with the exposure variable and the dependent variable, the latter causally. This distorts the relationship between the exposure and dependent variable unless taken into account by retaining them in the regression model [6]. Conversely, intermediate variables are on the causal pathway between exposure and dependent variable and the effect of the dependent variable is mediated in part or completely through the intermediate variable. Leaving intermediate variables within a regression model distorts the relationship between the exposure and the dependent variable towards the null [31]. In the model, confounding variables were retained if the adjusted estimate of the effect of MAP status differed by more than 10% from the crude estimate excluding the confounder. Where the causal web suggested that variables may be acting as partial intermediaries between MAP status and milk production, the direct effect of MAP status on milk production was also estimated by excluding the intermediary variables from the model [32, 33]. In addition, variables initially excluded with *p* < 0.1 under ANOVA pairwise comparison were reintroduced to check if they were confounders within the model. All regression models were tested using standard diagnostic techniques for homoscedasticity, normality of residuals, linearity of predictor-outcome association and the effect of outliers. Statistical analysis was performed using the statistical programme R v3.3.2 [34].

## Results

### Effect of JD status on milk production

Records from 259 cows were discarded (128 from 2013 to 14 and 131 from 2014 to 15) due to missing herd test data. Analysis of the missing data using the R package “MissMech” indicated that the missing data were not normally distributed (Hawkins *p* value < 0.001) but there was no evidence the missing data were heteroscedastic (non parametric test of homoscedasticity 0.427). Consequently, we concluded that the missing data were not normally distributed but there was no evidence they were not missing completely at random [35]. Subsequently, these missing data were excluded from analysis.

Across both seasons complete production records were available from 1122 cows in 2013–14 and 1069 cows in 2014–15. At the end of the 2013–14 season (May 2014), 388 cows were culled (114 with an elevated ELISA and/or fPCR status) and 335 heifers joined the herd in July 2014. Thus, of the 2191 lactation records with a known ELISA status, 1468 were from cows present in both years, 388 from cows present only in 2013–14 and 335 from cows present only in 2014–15. Of these ELISA positive animals, 405 had a known fPCR status. Test results and herd descriptive data are presented in Table 2, while descriptive data by category of MAP infection are presented in Table 3. Centiles and non-parametric test results are presented where the data distribution was not normal.

**Table 2:** Milk solids production, age and days in milk for cows which underwent screening for MAP infection

Season	2013–14			2014–15			*p*-value
Variable (Centile)	10th	50th	90th	10th	50th	90th	
Age (Years)	3	5	8	3	5	8	0.465^a^
Days in Milk	215	258	268	219	255	267	< 0.001^a^
Milk Solids (kg/cow)	307	433	552	337	480	590	< 0.001^a^
ISCC (cells/mL)	31 k	87 k	400 k	25 k	53 k	197 k	< 0.001^a^
Proportion Friesian	0.50	0.75	1.00	0.50	0.75	1.00	0.860^a^
Cows in milk	1122			1069			
ELISA Status	Number (Proportion)			Number (Proportion)			
Not Detected	825 (0.74)			961 (0.90)			< 0.001^b^
Low	157 (0.14)			39 (0.04)			< 0.001^b^
Moderate	63 (0.06)			27 (0.03)			< 0.001^b^
High	77 (0.07)			42 (0.04)			0.002^b^
Total	1122			1069			
fPCR Status of ELISA positive animals							
Not Detected	235 (0.79)			80(0.74)			0.557^b^
Moderate	32 (0.11)			11 (0.10)			0.865^b^
High	30 (0.10)			17 (0.16)			0.351^b^
Total	297			108			

^a^Wilcoxon Rank Sum

^b^χ^2^ test with Holme-Bonferonni correction

**Table 3:** Descriptive data by MAP infection status

MAP infection status	Average DIM (SD)	Average daily milk production in kgMS (SD)	Cows in category (proportion)	Average age (SD)	Total milk test results	First lactation results
Undetected	283 (19)	1.78 (0.58)	1779 (0.81)	5.0 (2.0)	7067	1828
Moderate latent	278 (22)	1.77 (0.59)	192 (0.09)	5.7 (1.7)	761	52
High latent	282 (18)	1.75 (0.49)	130 (0.06)	5.7 (1.9)	518	40
Moderate shedding	282 (18)	1.66 (0.53)	43 (0.02)	4.7 (1.5)	172	32
High shedding	282 (18)	1.59 (0.58)	47 (0.02)	5.5 (1.7)	188	24
Total	283 (19)	1.77 (0.58)	2191	5.2 (2.0)	8706	1976

Looking at the results for the ELISA (Paralisa™) test rather than the combination of ELISA and IDEXX tests used for classification in Table 2, in 2010–11 97/1086 (8.9%) animals were positive, compared to 365/1122 (33.0%) ELISA (Paralisa™) test positive animals in 2013–14 indicating that the prevalence of JD had continued to increase in this herd. In 2014–15 59/1069 (5.5%) were positive to the ELISA (Paralisa™) test. The change in prevalence of JD in this herd will be the subject of a separate study.

The effect of MAP infection status on milk production was confounded by age, season, days in milk and breed. However, the coefficients for the effect of MAP status changed by < 10% when somatic test score was included in the model and the impact of these changes in milk production was slight (+/− 0.01 kgMS). Consequently, LS was adjudged to be acting as an intermediary variable in this dataset and was excluded from the model [6].

The random effect of cow was significant in the final model (*p* < 0.001). The coefficients for the final mixed model illustrating the effect of MAP infection status on test day milk production for both years are presented in Table 4. The coefficients represent the effect of a unit change in the predictor variable on the predicted daily milk yield in kgMS given that all the other variables in the model do not change. There were no significant interactions between any of the variables in the model although there was a trend for the effect of MAP infection status on milk production to be greater in 2014–15 than 2013–14 (*p* = 0.065). However, the AIC value for the model with interaction were 10,205 with 20 degrees of freedom compared to 10,206 and 16 degrees of freedom for the model without interaction. Correspondingly the Bayesian Information Criterion (BIC) was 10,346 for the model with interaction and 10,319 for the simple model. The slight decrease in AIC indicated relatively little danger of underfitting in the simple model, while the larger increase in BIC suggested more danger of over fitting if the interaction was included [36]. Thus, coupled with a non-significant interaction term at the expense of increased model complexity and degrees of freedom the interaction term was not considered worthwhile for this data set.

**Table 4:** Results of a linear mixed model predicting daily kgMS production

Input variable	Coefficient	SE	*p*-value
Intercept	1.77	0.03	< 0.001
Parity			
Primiparous	Ref		
Multiparous	0.73	0.03	< 0.001
Days in Milk	−0.002	0.0001	< 0.001
Wilmink’s correction^a^			
exp(− 0.1 × DIM)	−0.13	0.22	0.546
Cow breed			
> 75% Friesian	Ref		
50–75% Friesian,	−0.09	0.02	< 0.001
< 50% Friesian	−0.07	0.02	< 0.001
DIM × parity			
DIM × Primiparous	Ref		
DIM × Multiparous	−0.002	0.0002	< 0.001
Wilmink’s correction × parity			
exp(−0.1 × DIM × primiparous)	Ref		
exp(−0.1 × DIM × multiparous)	−0.21	0.24	0.379
Season			
2013–14	Ref		
2014–15	0.22	0.01	< 0.001
MAP status^b^			
Undetected	Ref		
Moderate latent	−0.03	0.02	0.136
High latent	−0.07	0.03	0.004
Moderate shedding	−0.07	0.04	0.036
High shedding	−0.20	0.04	< 0.001

Ref = the reference category for each categorical variable

^a^Overall significance of Wilmink’s correction and its interaction in the model < 0.001

^b^Overall significance of MAP status in the model < 0.001

In each season the regression model predicted that after adjusting for the effects of age, days in milk, season and breed, cows that had a latent infection status or were shedding MAP produced less milk than cows where MAP was not detected (not significant for moderate latent cows). Tests of the significance of the effects of differing MAP infection status on milk production with Tukey contrasts for multiple comparisons are shown in Table 5.

**Table 5:** Comparison tests for the effect of MAP infection status on daily milk production. *P*-values are Tukey contrasts for multiple comparison

Comparison	Difference	SE	*p*-value
Significant differences			
High latent vs negative 2–0	− 0.07	0.03	0.004
Moderate shedding vs negative 3–0	− 0.07	0.03	0.036
High shedding vs negative 4–0	− 0.20	0.04	< 0.001
Moderate latent vs high shedding 1–4	+ 0.17	0.05	0.002
High latent vs high shedding 2–4	+ 0.13	0.05	0.050
Non-significant differences			
Moderate latent vs negative 1–0	−0.03	0.02	0.136
Moderate latent vs high latent 1–2	+ 0.04	0.03	0.679
Moderate latent vs moderate shedding 1–3	+ 0.04	0.05	0.890
Moderate shedding vs high shedding 3–4	+ 0.12	0.06	0.194
High latent vs moderate shedding 2–3	−0.00	0.05	1.000

Generally, all infection statuses were associated with reduced milk production compared to cows where MAP was not detected (not significant for moderate latent cows). There was no difference within the categories of shedding nor within the categories of latency. Moderate and high shedders produced less milk than all other categories but this effect was only significant for high shedding cows. The model predicted milk solid production at the average number of days in milk (141 days) by MAP status together with pairwise comparison with Tukey adjusted *p*-values is shown in Table 6.

**Table 6:** Model predicted daily milk production (kgMS/day) at different MAP infection status. *P*-values are Tukey contrasts for multiple comparison

infection status	Predicted daily milk production and 95% CI (kgMS/day)	Significance of Tukey adjusted pairwise comparison of MAP status^a^.
Undetected	1.74 (1.71–1.76)	b
Moderate Latent	1.71 (1.66–1.77)	b
High Latent	1.67 (1.60–1.73)	b
Moderate Shedding	1.64 (1.56–1.72)	ab
High Shedding	1.53 (1.42–1.63)	a

^a^Values with different letters are significantly different at *p* < 0.05

**Fig. 1:**
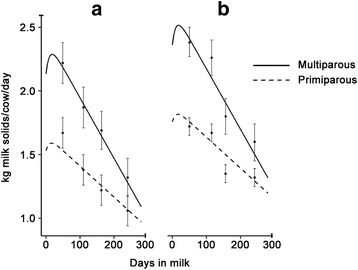
Predicted milk yield for multiparous and primiparous cows in 2013–14 (**a**) and 2014–15 (**b**) from a model investigating the effect of MAP infection status on milk yield. Ninety-five percent confidence intervals for the observed milk yield at each of four test dates in each lactation are represented by vertical lines in each lactation

A smoothed plot of the predicted milk solids compared with the observed average milk solids and the 95% confidence interval for the range of observed milk solids at each herd test is depicted in Fig. 1 to demonstrate that the model adequately predicted the observed lactations. Ninety-five percent confidence intervals for the observed milk yield at each of four test dates in each lactation are represented by vertical lines in each lactation.

## Discussion

This study supports the hypothesis that sub-clinical MAP infection reduces milk production. To our knowledge this is the first longitudinal study based in NZ looking at the effect of MAP infection status on daily milk production while allowing for intermediary and confounding factors.

We recognize that our interpretation of these results is based on an imperfect testing methodology in that neither test had 100% sensitivity and specificity nor were all cows tested with both ELISA and fPCR. Nevertheless, in a single herd study, Aly et al. [37] identified that qPCR testing of faecal samples from ELISA positive cows in high MAP bioburden pens was the most cost effective strategy for confinement systems. Whilst pen sampling is not applicable in pasture systems, these workers also suggested that whole herd milk or single antigen ELISA serum test followed by fPCR on ELISA positive cows was the next most cost effective strategy and increased sensitivity to 68.4%.

O’Brien et al. [26] described a synergistic effect through the incorporation of a dual ELISA tests which resulted in an 18 and 17% increase in overall test sensitivity over individual tests used in isolation (IDEXX and Paralisa™, respectively) for defined fecal shedding states in a dataset comprising 1069 matched bovine fecal samples submitted for routine testing. These authors reported a sensitivity of the composite ELISA tests of 92%, with a specificity of 59% for detection of ≥1 × 10^3^ MAP genomes/mL based on this matched dataset. These authors acknowledged that while it was possible that the increased sensitivity observed through the inclusion of additional antigens arose at the cost of test specificity, true specificity values could not be derived from the dataset as the samples were submitted as part of routine testing from a number of infected herds. It is vital to maximise the sensitivity of the initial screening test to identify the maximum numbers of putative shedders. While Aly et al., [37] suggest that specificity is the most important parameter in developing a cost effective strategy to control MAP infection, for serial test systems it is the specificity of the composite tests (ELISA + fPCR) rather than individual test results that are critical.

The ELISA screening test was designed to have maximal sensitivity to select a group of animals at risk of shedding, from which a subgroup of high shedders were identified with the confirmatory fPCR test which has a specificity of virtually 100% [37]. Using these figures, a priori*,* we believed our combined ELISA test had a sensitivity of 0.92 and a specificity of 0.81 which would suggest that 21% would test positive (true and false positives). In total we tested 2191 cows and found 19% were test positive.

Although the results are derived from one herd over a two-year period and so cannot be readily extrapolated to other NZ dairy herds, they are in line with the effect of MAP infection outlined by others although differences in testing methodology make direct comparison difficult. Using a single serum ELISA test and fecal culture where > 30 MAP cfu/g feces was classified as high shedding, Smith et al. [4] reported a reduction in daily milk production equivalent to around 0.2–0.3 kgMS. Using a similar testing methodology, Aly et al. [6] reported that fecal positive cows produced the equivalent of 0.2 kgMS less per day compared to cows where MAP was not detected. Smith et al. [4] reported that latent infected animals produced an equivalent 0.2 kgMS/day more milk than negative cows and they postulated a positive genetic association between susceptibility to MAP infection and milk production. There was no positive association between latent status and milk production in the current study; moderate latent cows had no significant difference in production compared to cows where MAP was not detected and high latent cows produced 0.07 kgMS/day less. Smith et al. [4] found that, although latent cows produced more milk than cows where MAP was not detected, the difference decreased with time spent in the latent infection state. In the present study, cows were tested once near the end of lactation and so the tested population may represent cows more advanced in the infection process than when testing is quarterly.

Other studies have also identified a positive or null association between MAP infection and milk production [38, 39]. Johnson et al. [40] found no effect on milk production of subclinical JD infection (as diagnosed by serum ELISA and fecal radiometric culture). The authors of these studies suggested that the inconsistency reported between different studies in the effect of MAP status on milk production is because the effect of MAP status depends upon the parity of the cow [40] or the production potential [41] and that younger, more productive cows showed a positive association between MAP status and milk production whereas older cows only showed a negative association between MAP status and milk production in the lactation before culling. However, the herd age distribution in these studies was considerably younger than in the current herd with Johnson et al. [40] reporting 59% of the study herds in their first or second lactation and the average age at 3.67 years. In the present study, 45% of animals were in their first or second lactation and the average age was 5.2 years. These workers also found no difference in the prevalence of MAP positive cows with age whereas in this NZ study, prevalence of MAP infection increased with age.

In the present study, latent and shedder status were subdivided to see if differences in the cow’s response to MAP infection (from whatever cause) was reflected in a measurable production response. The strength of the ELISA response has been predictive of the decrease in milk production in other studies [42] and has been recommended as a factor to guide culling decisions [43].

In both their early study and a later follow up, Smith et al. [4, 5] were able to look at the effect of changes in MAP infection status as cows were sampled multiple times during the lactation. These authors found that some infected animals went on to become high shedders (> 50 cfu/g) while others remained low shedders for the duration of the study (7 years). Although both groups suffered a drop-in production, this partially recovered in some low shedding cows.

Smith et al. [5] found that the number of fecal positive tests was a good indicator of the effect on milk production; cows with multiple fecal positive test results were more likely to have a progressing infection and ongoing decreased milk production. As these authors acknowledge, multiple tests are likely to prove cost prohibitive on commercial dairy farms. However, the quantitative fPCR used in the present study may offer an advantage in allowing classification and stratification of shedding animals. The present study cannot determine how the screening test results used relate to the infection path described by Smith et al. [5] but they indicate that, based on the differences in predicted daily milk solids production over a 282 day lactation, high shedding animals have a more dramatic reduction in milk production (− 59.22 kgMS over 282 days lactation) than low shedders (− 28.20 kgMS over 282 days lactation). Knowledge of the average, predicted drop in milk solids can help farmers to prioritize animals for culling although a thorough evaluation of the most appropriate control strategy must consider the number of cows in each MAP class as well as their fecal MAP burden.

Fecal shedding in infected animals had a consistently more negative effect on production than latent infection as has been found in other studies [3]. However, as these workers point out this may be due to differences in test specificity rather than to differences in pathology. With a specificity of 59–81% for the pooled ELISA and close to 100% for the fPCR [37], more false positive cows will be identified by the ELISA test than the fPCR. These MAP negative cows would decrease the apparent effect of a positive ELISA status on milk production.

The present results may have been biased by the loss of animals with incomplete records, especially if these were low producing animals, culled before the autumn MAP test. However, the loss of these cows would have biased the results towards non-significance [4]. Similarly, lack of sensitivity in any of the single ELISA tests would have had the same effect by failing to detect MAP infected animals but this was offset by using multiple ELISA tests in parallel. In the current study, fecal shedders were classified as any cow that had a positive fPCR whereas Smith et al. [4], with a single ELISA classification, defined all ELISA positives as fecal shedders. In the present study, with the reduced specificity from ELISA tests in parallel, classifying all ELISA positive cows as fecal shedders would have led to false positives. We believe that our parallel ELISA testing maximises sensitivity and so reduces the number of false negatives that go forward to fPCR testing. The greater sensitivity of fPCR compared to fecal culture [19] then supports the contention that the risk of failing to identify shedders in this group was low. Although fecal contamination during collection of fecal samples could lead to false positive fecal results the quantitative nature of fPCR means that it is unlikely that this would be sufficient to change the fPCR shedding classification of the cow. Conversely, while tissue and for many practical purposes, fecal culture on solid medium remains the gold standard for specificity, the long incubation period required means that this test may be compromised with overgrowth of contaminating gut bacteria [24]. Sensitivity of fecal culture in high prevalence herds has been estimated at 53% for sub-clinical cows [44].

We found no interaction between age and MAP infection status (*p* > 0.1) although numerically suggestive of an increase in effect with age as described by others [6, 22, 45]. The small sample size in the current study and the relative lack of MAP positive 2 year olds may have meant there was insufficient power to detect such a relationship at a statistically significant level. In pastoral systems, matching of feed supply to cow demand is highly dependent on seasonal factors [46] and so the ability of cows to milk to their potential is variable year to year. This is consistent with differences in the degree to which MAP status impacts on milk production suggested by the statistical trend for an interaction between season and MAP status in this model. This contrasts with models from confined all year round calving systems [4, 5] where no such interaction was present but where feed supply-cow demand is much less influenced by seasonal climate. However, others have suggested that interaction terms should be interpreted more conservatively (*p* < 0.001) to avoid Type 1 errors and so we elected to use the simpler model without interaction [47].

In this study we describe the application of DNA standards to facilitate quantitative judgements of MAP shedding. These standards spanned the range of MAP shedding observed in clinical samples by the testing laboratory using this approach and were linear in the assay over the 7 logs (typically r^2^ = 0.999). Although detection of MAP DNA at lower titers is readily achievable, sample replicates may become poor because of stochastic variation [20] and, in JD affected herds, extremes of diagnostic sensitivity for MAP bacilli in feces may be of dubious clinical relevance or may be otherwise attributable to pass-through shedding in a contaminated environment; for these reasons a conservative lower detection cut point was favored. While there is surprisingly little agreement on standardization of fPCR data for quantitative reporting of MAP fecal shedding [47] in this study DNA standards were favored for quantitation as they are quick and inexpensive to prepare and to quantify accurately and also stable in storage, allowing a set of quantitation standards to be prepared that are highly reproducible from day to day and which facilitate objective comparisons between samples. More importantly they are constant, comparable and reproducible across laboratories, geography and time.

While recommendations for optimal diagnostic strategies can be made for NZ informed by international best practice and studies performed overseas, they must also be considered in the light of cost and local availability of diagnostic services. Due to the extreme demands placed on diagnostic tests by the dynamics of MAP infection and the persistent, chronic nature of JD progression in cattle, single, non-quantitative tests used in isolation may be insufficiently exact to maximally inform management decisions particularly in the early, subclinical stages of disease when bioindicators are absent. Diagnostic tests based on multiple ELISA and fPCR are quick, inexpensive and quantitative while also amenable to the incorporation of additional antigens to broaden their diagnostic repertoire at little additional cost.

## Conclusion

In this herd we identified a significant effect of subclinical MAP infection on milk solids production. The testing methodology used in the present study allowed the farmer to identify cows that were infectious and our model suggests they are likely to under produce compared to cows where MAP is not detected. Although our findings are from a single herd study and cannot be extrapolated to the wider farming environment, they are in line with similar studies involving small numbers of herds [4, 37].

Greater knowledge allows better decisions to be made about which animals to cull to both reduce the infectious pressure within the herd and the production losses associated with infection. Early culling of sub-clinically affected animals can be justified both on epidemiological grounds and because of the associated deficits in production. While there is contention as to the impact and costs associated with subclinical MAP infection in dairy cows, the deficits in milk production seen in the current study suggest that composite diagnostic testing to identify and cull fPCR shedders and high latent infected cows may be justified.

## Abbreviations

DIM: Days in milk
DRL: Disease Research Ltd
EU: ELISA units
fPCR: Fecal PCR
ISCC: Individual somatic cell count
JD: Johne’s disease
LS: Somatic cell linear score
MAP: *Mycobacterium avium* subsp. *paratuberculosis*
NZ: New Zealand
PPA: Protoplasmic antigen
